# Synthesis of Bis{*meso*-Tetrakis(4-*N*-alkylpyridiniumyl)porphyrinato}cerium and Its Redox Switching Behavior †

**DOI:** 10.3390/molecules26040790

**Published:** 2021-02-03

**Authors:** Toshio Nishino, Yasuyuki Yamada, Ayumi Yamamoto, Kentaro Tanaka

**Affiliations:** 1 Department of Chemistry, Graduate School of Science, Nagoya University, Furo-cho, Chikusa-ku, Nagoya 464-8602, Japan; t-nishino@ms.naist.jp (T.N.); yamada.yasuyuki@h.mbox.nagoya-u.ac.jp (Y.Y.); yamamoto.ayumi@a.mbox.nagoya-u.ac.jp (A.Y.); 2 Research Center for Materials Science, Nagoya University, Furo-cho, Chikusa-ku, Nagoya 464-8602, Japan; 3 JST, PRESTO, 4-1-8 Honcho, Kawaguchi, Saitama 332-0012, Japan

**Keywords:** double-decker cerium complex, octacationic complex, redox switching

## Abstract

A novel double-decker porphyrin complex, bis{*meso*-tetrakis(4-*N*-alkylpyridiniumyl)porphyrinato}cerium, was prepared. Electrochemical measurements revealed that this complex exhibited reversible redox waves corresponding to a 1e^–^ redox reaction of the cerium center. Treating the complex alternately with an oxidant and a reductant resulted in the reversible redox switching between the oxidized and reduced states in an organic solvent.

## 1. Introduction

**Figure 1:**
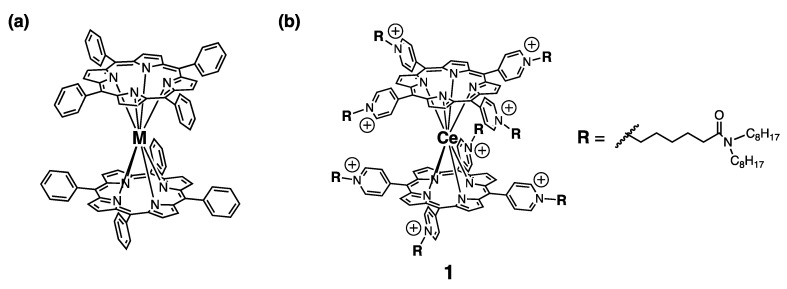
(**a**) Double-decker porphyrin complex and (**b**) Bis{*meso*-tetrakis(4-*N*-alkylpyridiniumyl)porphyrinato}cerium (**1**) prepared in this work.

Double-decker porphyrinoid complexes, in which a large metal ion such as a lanthanoid ion is sandwiched between two porphyrinoid compounds (Figure 1a), have attracted significant interest from chemists since the first report on the bis(phthalocyaninato) complex in the 1960s [1,2]. A particularly interesting aspect of these complexes is that an appropriate combination of the metal ion and porphyrinoids can generate intense π–π and metal–π interactions, resulting in unique molecular properties such as multiple redox properties [3,4,5,6,7], single molecular magnetism [8,9,10,11,12,13,14,15,16], and organic field effect transistor properties [17,18,19]. Furthermore, the unique double-decker structure inspired chemists to utilize these complexes as the key components of molecular machines [20,21,22,23] and molecular receptors [24,25,26,27], and as skeletons for specific molecular architectures [28,29,30,31,32]. Therefore, the synthesis of such double-decker porphyrinoid complexes with novel structures can contribute to the construction of a rich library of the corresponding chemical modules.

Since the physical properties of the double-decker porphyrinoid complexes are drastically modulated by the combination of the central metal ion and porphyrinoids, systematic investigations are necessary to clarify the relationship between their optical, redox, and electronic properties and their chemical structures. Herein, we report the synthesis and electrochemical properties of a novel bis(porphyrinato)cerium complex (Figure 1b), bis{*meso*-tetrakis(4-*N*-alkylpyridiniumyl)porphyrinato}cerium (**1**). Tetrapyridiniumporphyrins are a series of the most common water-soluble tetracationic porphyrins. However, there are only a few reports on the synthesis or detailed redox properties of double-decker complexes using tetrapyridiniumporphyrins as ligands [33,34,35,36,37,38,39]. We speculated that the introduction of long-chain alkyl moieties to the pyridinium unit would improve the solubility in various organic solvents. Improving the solubility will make this complex more tractable and enable its detailed characterization. Moreover, since the eight peripheral pyridinium units are expected to affect the physical properties of the bis(porphyrinato)cerium core, it will be interesting to investigate the detailed redox and optical properties of **1**.

## 2. Results and Discussion

**Scheme 1:**
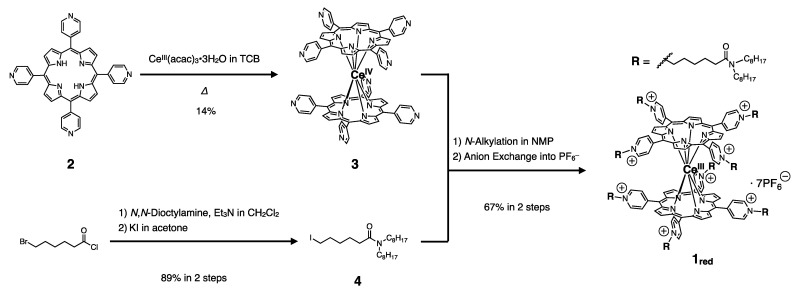
Synthesis of reduced complex **1_red_**.

**Figure 2:**
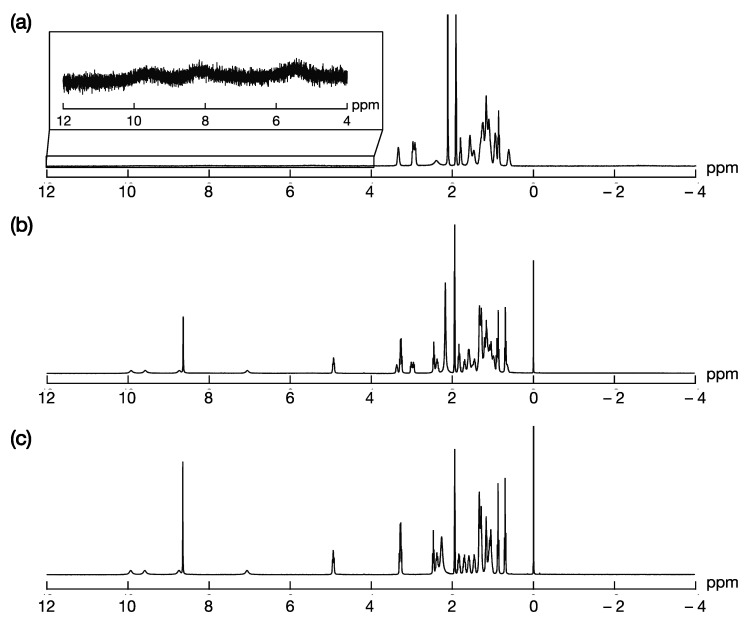
^1^H NMR titration of **1_red_** (2.5 mM in CD_3_CN) with NO^+^·PF_6_^−^. ^1^H NMR spectra of (**a**) **1_red_**, (**b**) a mixture of **1_red_** and 0.5 equiv. of NO^+^·PF_6_^−^, and (**c**) a mixture of **1_red_** and 1.0 equiv. of NO^+^·PF_6_^−^.

We first prepared bis(tetra(4-pyridyl)porphyriato)cerium(IV) (**3**) by refluxing a solution of Ce(acac)_3_·*n*H_2_O with tetra(4-pyridyl)porphyrin in 1,2,4-trichlorobenzene (isolated yield: 29%) as shown in Scheme 1. Cerium was determined to be tetravalent in **3** based on its sharp ^1^H NMR signals (Figure S1), suggesting that the cerium ion was oxidized by air during purification. Similar oxidation of the cerium center in double decker complexes has been reported previously by some groups [34,35]. Complex **3** was then functionalized with alkyliodide (**4**), followed by counter anion exchange to give the desired double decker complex with octapyridinium pendants, **1_red_**, in 67% isolated yield. Interestingly, compared to the ^1^H NMR signals of **2**, the ^1^H NMR signals of isolated **1_red_** were significantly shifted and broadened, especially those corresponding to the aromatic groups of the porphyrin moieties (Figure 2a). This suggested the reduction of the cerium center during the introduction of the alkyl chains. We speculated that the cerium center in **1** was reduced by the I^−^ ion generated during the nucleophilic attack of **3** on iodoalkane **4**.

**Figure 3:**
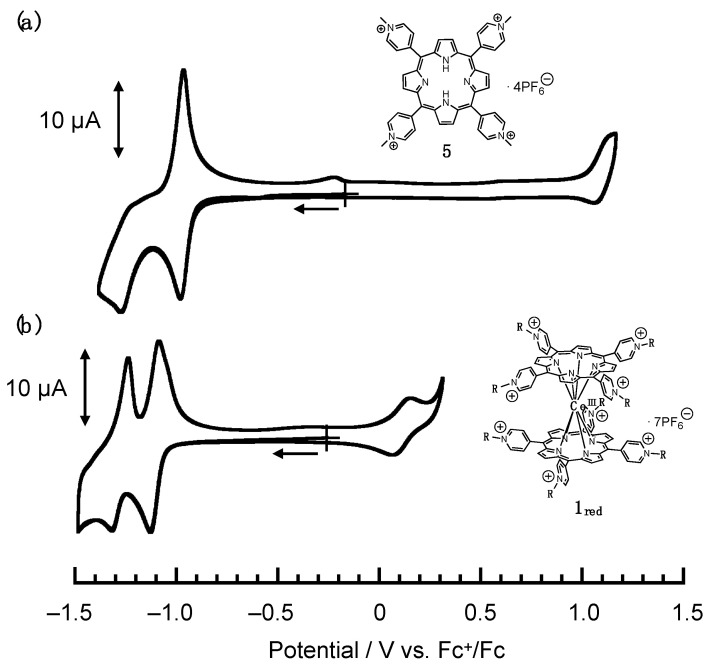
Cyclic voltammograms of (**a**) **5** and (**b**) **1_red_** in CH_3_CN solution containing 100 mM *n*Bu_4_NPF_6_. Measurements were performed at a scan rate of 100 mV/s at 20 °C; [Substrate] = 300 μM.

Cyclic voltammograms of **1_red_** in a CH_3_CN solution containing 100 mM *n*Bu_4_N^+^PF_6_^−^ was compared with that of the monomeric reference, 5,10,15,20-tetrakis(*N*-methylpyridinium-4-yl)porphyrin·4PF_6_^−^ (**5**) (Figure 3). The large redox waves of **1_red_** at −1.11 and −1.28 V vs. Fc^+^/Fc are assignable to the peripheral pyridinium ions, based on the similar redox peaks observed for **5**. Considering that the redox potential of the porphyrin center in **5** was higher than 1.0 V, a reversible redox wave at 0.11 V could be assigned to the Ce^4+^/Ce^3+^ conversion in **1**. This Ce^4+^/Ce^3+^ redox wave for **1** showed a significant positive shift with respect to that for bis(tetraphenylporphyrinato)cerium (*E*°(Ce^4+^/Ce^3+^) = −0.36 V vs. Fc^+^/Fc) [40]. This is presumably due to the effect of the positively charged pyridinium moieties on the ligands. The fact that the rest potential of the solution of **1_red_** subjected to cyclic voltammetry is around −0.26 V, which is much more negative than the *E*°(Ce^4+^/Ce^3+^) value of **1** (0.11 V), clearly indicates that the cerium center in **1** is in the Ce(III) state.

Considering that the 1e^–^ oxidation of the cerium center in **1_red_** is reversible, we next attempted the redox switching of the cerium center in **1**. Addition of an increasing amount of NO^+^·PF_6_^−^, a strong oxidizing reagent, in a CD_3_CN solution decreased the intensities of the signals corresponding to **1_red_**, while the intensities of the sharp signals corresponding to the oxidized form of **1** (**1_ox_**) increased (Figure 2). After the addition of one equivalent of NO^+^·PF_6_^−^, the signals corresponding to **1_red_** completely disappeared, and **1_ox_** was quantitatively generated. We washed the CHCl_3_ solution of **1_ox_** with an aqueous solution containing excess KI, and found that **1_red_** was quantitatively obtained as described in the experimental section. Thus, reversible switching of the redox state, i.e., reversible conversion between **1_red_** and **1_ox_**, was achieved by treating the sample alternately with NO^+^·PF_6_^−^ and KI.

**Figure 4:**
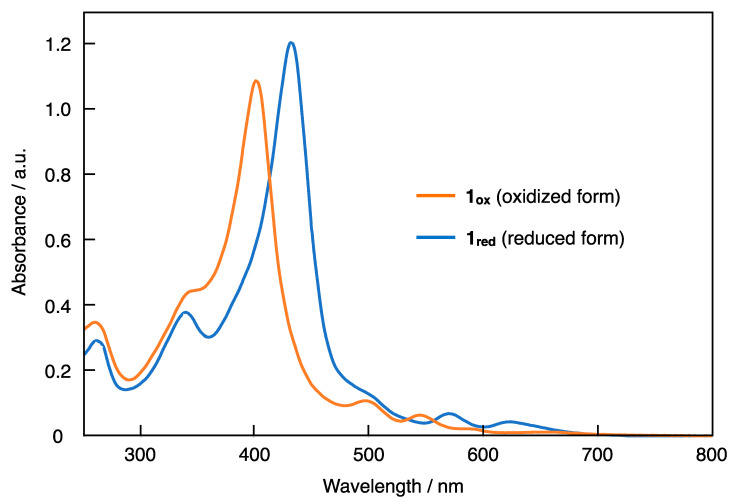
Comparison of UV–VIS spectra of **1_red_** and **1_ox_**.

The UV–VIS spectra of **1_ox_** and **1_red_** in CH_3_CN solution are shown in Figure 4. Complex **1_ox_** showed a characteristic Soret band at 401 nm and Q bands at 497, 545, 588, and 656 nm, which were almost identical to those of bis(tetrapyridylporphyrinato)cerium(IV) (Soret band: 398 nm, Q-bands: 490, 543, 588, and 645) [36]. On the other hand, **1_red_** showed the Soret band at 432 nm and the Q bands at 502, 570, 623, and 680 nm. Thus, there were significant changes in the absorption spectra due to the change in the redox state of the cerium center. This behavior was similar to that observed for the redox change of bis[tetrakis(4-*N*-methylpyridiniumyl)porphyrinato]cerium(III) heptachloride ([Ce^III^(TM_4_PyP)_2_]Cl_7_) in an aqueous solution, as reported by Nawra et al. [36].

## 3. Materials and Methods

### 3.1. General Information

The synthetic procedures were carried out under an anhydrous nitrogen atmosphere, unless otherwise specified. All the reagents and solvents were purchased at the highest commercial quality available and used as received without further purification, unless otherwise stated. The ^1^H, and ^13^C NMR spectra were recorded using a JEOL JNM-ECS400 (400 MHz for ^1^H; 100 MHz for ^13^C) or a JEOL JNM-ECA600 (600 MHz for ^1^H; 150 MHz for ^13^C) spectrometer at a constant temperature of 298 K. Tetramethylsilane (TMS) was used as the internal standard for the ^1^H and ^13^C NMR measurements in CDCl_3_, CD_3_CN, and DMSO-*d*_6_. The silica gel column chromatographies and thin-layer chromatography (TLC) were performed using Merck silica gel 60 and Merck silica gel 60 (F254) TLC plates, respectively. The ESI mass spectrometry was performed using a Waters LCT-Premier XE spectrometer, controlled using the Masslynx software. The absorption spectra were recorded using a Hitachi U-4100 spectrophotometer in CHCl_3_ solutions at 20 ± 0.1 °C in quartz cells (10 mm optical path length). Cyclic voltammetry measurements were performed with a BAS Electrochemical Analyzer Model 750Ds at room temperature, in acetonitrile solutions containing 0.1 M tetrabutylammonium hexafluorophosphate (TBAPF_6_) in a standard one-component cell under an N_2_ atmosphere equipped with a 3 mm-O.D. glassy carbon disk working electrode, and a platinum wire counter electrode as Ag/Ag^+^ reference electrode. All solutions were deoxygenated by N_2_ bubbling for at least 10 min. Obtained *E*^0^^′^ vs. Ag/Ag^+^ were converted to those vs. Fc^+^/Fc based on measured redox potential of ferrocene.

### 3.2. Synthesis

*N*,*N*-Di-*n*-octyl-6-bromohexanamide

A mixture of di-*n*-octylamine (7.6 mL, 25 mmol) and Et_3_N (4.2 mL, 30 mmol) in CH_2_Cl_2_ (80 mL) was added dropwise to a CH_2_Cl_2_ solution (400 mL) containing 6-bromohexanoyl chloride (4.6 mL, 30 mmol) for 4 h at 0 °C. The resulting solution was stirred for further 30 min at 0 °C. H_2_O (20 mL) was added to the reaction mixture. The separated organic phase was washed with 1 M aqueous H_2_SO_4_ (150 mL), saturated aqueous NaHCO_3_ (300 mL), H_2_O (300 mL), and brine (150 mL × 3), then dried over anhydrous Na_2_SO_4_, filtrated, and evaporated to afford a colorless oil (14.9 g). The crude compound was purified by silica gel column chromatography (*φ* 5 × 11 cm, hexane:AcOEt = 9:1) to afford the title compound as a colorless oil (10.2 g, 97%). ^1^H NMR (400 MHz, CDCl_3_/TMS): *δ* = 3.42 (t, *J* = 6.8 Hz, 2H), 3.28 (t, *J* = 7.7 Hz, 2H), 3.19 (t, *J* = 7.8 Hz, 2H), 2.30 (t, *J* = 7.4 Hz, 2H), 1.89 (tt, *J* = 7.1, 7.1 Hz, 2H), 1.67 (tt, *J* = 7.6, 7.6 Hz, 3H including H_2_O), 1.54–1.48 (m, 6H), 1.28 (br, 20H), 0.91–0.86 (m, 6H). ESI-TOF MS (positive) *m/z* = 418.2 [M + H]^+^, 418.1 calced [M + H]^+^.

*N*,*N*-Di-*n*-octyl-6-iodohexanamide (**4**)

A mixture containing *N*,*N*-di-*n*-octyl-6-bromohexanamide (10.2 g, 24 mmol) and NaI (11.2 g, 75 mmol) in acetone (50 mL) was refluxed for 1 hr. The reaction mixture was evaporated and AcOEt (120 mL) was added to the residue. The solution was washed with H_2_O (100 mL × 2) and brine (100 mL × 3), then dried over anhydrous Na_2_SO_4_, filtrated, and evaporated to afford a crude oil. The crude compound was purified by silica gel column chromatography (*φ* 5 × 16 cm, hexane:AcOEt = 19:1–9:1) to afford the title compound as a pale yellow oil (10.2 g, 92%). ^1^H NMR (400 MHz, CDCl_3_/TMS): *δ* = 3.28 (t, *J* = 7.6 Hz, 2H), 3.22–3.17 (m, 4H), 2.30 (t, *J* = 7.4 Hz, 2H), 1.86 (tt, *J* = 7.4, 7.4 Hz, 2H), 1.67 (tt, *J* = 7.7, 7.7 Hz, 2H), 1.56–1.44 (m, 6H), 1.28 (br, 21H), 0.91–0.86 (m, 6H). ESI-TOF MS (positive) *m/z* = 466.2 [M + H]^+^, 466.3 calced [M + H]^+^.

**1_red_**

A mixture of bis(tetra(4-pyridyl)porphyrinato)cerium(IV) (27 mg, 19.4 µmol) and *N*,*N*-di-*n*-octyl-6-iodohexanamide (706 mg, 1.5 mmol) in NMP (0.5 mL) in a Schlenk flask was heated at 100 °C for 90 hrs. The reaction was monitored by ESI-TOF mass spectroscopy. The reaction mixture was poured into H_2_O (150 mL). The residual black oil was collected by decantation. After the mixture was dissolved in CH_2_Cl_2_ (100 mL), the solution was dried over anhydrous Na_2_SO_4_, filtrated, and evaporated. Hexane (100 mL) was added to the residue. The resulting precipitate was filtrated (114 mg). The solid residue was dissolved in a 1:1 mixture (*v*/*v*) of CHCl_3_ and MeOH (20 mL), and then, ion-exchange resin (IRA400J CL (Cl^-^ form)) (20 mL) was added. The resulting mixture was kept standing at room temperature for 20 min and the resin was removed by filtration. This ion-exchange procedure was repeated twice to afford a crude solid (964 mg). The crude compound was purified by silica gel column chromatography (4 cm *φ* × 20 cm, CH_2_Cl_2_:MeOH:1% aqueous NaCl = 5:1:0–2:1:0–2:1:0.03). The fraction eluted by a mixture of CH_2_Cl_2_:MeOH:1% aqueous NaCl = 2:1:0–2:1:0.03 was evaporated. The resulting residue was redissolved in CH_2_Cl_2_ (100 mL). The organic phase was washed with H_2_O (100 mL × 2) and brine (100 mL × 2), the dried over anhydrous Na_2_SO_4_, filtrated, and evaporated to afford blackish-brown solid (88 mg). The compound was dissolved in MeOH (15 mL) and a solution of KPF_6_ in CH_3_CN (3 mL) was added. Slow addition of H_2_O (10 mL) gave precipitate. This ion exchange procedure was repeated twice to afford the title compound as a blackish-brown solid (63 mg, 67%). ^1^H NMR (400 MHz, CD_3_CN/TMS): *δ* = 3.37–3.39 (br, 16H), 2.94–3.03 (m, 34H), 2.41–2.52 (br, 9H), 1.84 (t, *J* = 7.0, 15H), 1.54–1.65 (br, 49H), 1.40–1.05 (m, 186H), 0.97–0.99 (br, 35H), 0.90 (t, *J* = 26H, 25H,), 0.60–0.70 (br, 16H).

**1_ox_**

A 0.3 M NO^+^·PF_6_^–^ solution in MeCN (66.4 µL, 20 µmol) was added to a solution of **1_red_** (102 mg, 20 µmol) in MeCN (15 mL). The resulting mixture was evaporated and the residue was washed with H_2_O (10 mL) to obtain **1_ox_** as a blackish-brown solid (101 mg, 97%). ^1^H NMR (600 MHz, CD_3_CN/TMS): *δ* = 9.93 (br, 7H), 9.59 (br, 7H), 8.74 (br, 7H), 8.64 (s, 16H), 7.06 (br, 8H), 4.94 (t, *J* = 7.2 Hz, 16H), 3.30–3.26 (m, 32H), 2.47 (t, *J* = 7.2 Hz, 16H), 2.40–2.35 (m, 16H), 1.87–1.82 (m, 20H), 1.70 (tt, *J* = 7.5, 7.5 Hz, 16H), 1.59 (br, 16H), 1.46 (br, 16H), 1.37–1.27 (br, 94H), 1.16 (br, 32H), 1.10–1.00 (m, 48H), 0.87 (t, *J* = 7.2 Hz, 24H), 0.69 (t, *J* = 7.2 Hz, 24H). Anal. Calcd. for C_256_F_48_H_400_N_24_O_8_P_8_Ce (**1_red_**·8PF6): C, 58.56; H, 7.70; N, 6.40. Found. C, 58.66; H, 7.69; N, 6.41 (0.10% error).

Reduction of **1_ox_** into **1_red_** by treatment with aqueous KI

**1_ox_** (5.2 mg, 1.0 µmol) in CHCl_3_ (15 mL) was washed with 10% aqueous KI (10 mL × 2). The organic phase was dried over anhydrous Na_2_SO_4_, filtrated, and evaporated to obtain **1_red_** as a 7I^–^ salt (5.0 mg, quant.). ^1^H NMR (400 MHz, DMSO-*d*_6_/TMS): *δ* = 9.78 (br, 3H), 8.68 (br, 4H), 5.93 (br, 5H), 2.92 (br, 32H), 1.81 (t, *J* = 6.0 Hz, 16H), 1.32–1.11 (m, 178H), 1.02 (br, 36H), 0.94 (br, 28H), 0.86 (t, *J* = 6.2 Hz, 28H), 0.80 (t, *J* = 6.8 Hz, 24H), 0.62 (br, 16H), −2.82 (br, 5H).

## 4. Conclusions

We prepared a novel bis(porphyrinato)cerium(III) complex containing eight pyridinium moieties, **1_red_**, based on the peralkylation of a bis(tetrapyridylporphyrinato)cerium(IV) complex via the reaction with iodoalkane. The obtained double-decker complex, **1_red_**, included a Ce(III) core, implying that the cerium center underwent 1e^–^ reduction upon the reaction with I^−^ during the introduction of the peripheral alkyl chains, as confirmed by cyclic voltammetry and ^1^H NMR and UV–VIS spectroscopies. We demonstrated that the addition of an equimolar amount of an oxidant, NO^+^·PF_6_^−^, converted **1_red_** into its oxidized form **1_ox_** quantitatively. This suggested that the alternating addition of NO^+^·PF_6_^−^ and KI enabled reversible switching of the redox states of the cerium center in double-decker complex **1** in an organic solvent. As far as we know, this is the first example of a reversible redox switching of a bis(tetrapyridylporphyrinato)cerium complex in an organic solution. Reversible redox of the cerium center with apparent color change should be suitable for a colorimetric indicator of oxidants, such as halogen and nitrosonium ions. This would also suggest potential applications in redox switchable molecular devices.

## Data Availability

The data presented in this study are available on request from the corresponding author.

## Supplementary Materials

The following are available online at https://www.mdpi.com/1420-3049/26/4/790/s1, Synthesis of bis{*meso*-tetrakis(4-N-alkylpyridiniumyl)porphyrinato}cerium and its redox switching behavior, Figure S1: ^1^H-NMR spectrum of bis(tetra(4-pyridyl)porphyrinato)cerium(IV) **3** in CDCl_3_ at 20 °C.

## Sample Availability

Samples of the compounds are not available from the authors.
