# Supplementary material for: Synthesis of Bis{meso-Tetrakis(4-N-alkylpyridiniumyl)porphyrinato}cerium and Its Redox Switching Behavior
Source: Molecules. 2021 Feb 3;26(4):790. doi: 10.3390/molecules26040790 (PMC7913613; doi:10.3390/molecules26040790)
Supplement: Supplementary file 1 [file molecules-26-00790-s001.pdf]

Supplementary Information for

Synthesis of bis{*meso*-tetrakis(4-N-alkylpyridiniumyl)porphyrinato}cerium and its redox switching behavior

Toshio Nishino [1], Yasuyuki Yamada [1,2,3], Ayumi Yamamoto [1], and Kentaro Tanaka [1,*]

[1] Department of Chemistry, Graduate School of Science, Nagoya University, Furo-cho, Chikusa-ku, Nagoya 464-8602, Japan
[2] Research Center for Materials Science, Nagoya University, Furo-cho, Chikusa-ku, Nagoya 464-8602, Japan
[3] JST, PRESTO, 4-1-8 Honcho, Kawaguchi, Saitama, 332-0012, Japan

* E-mail: kentaro@chem.nagoya-u.ac.jp

Page S2: Synthesis of bis(tetra(4-pyridyl)porphyrinato)cerium(IV) **3**

Page S3: ^{1}H-NMR spectrum of bis(tetra(4-pyridyl)porphyrinato)cerium(IV) **3** in $CDCl_3$ at 20 °C. (*Figure S1*)

Bis(tetra(4-pyridyl)porphyrinato)cerium(IV) **3**

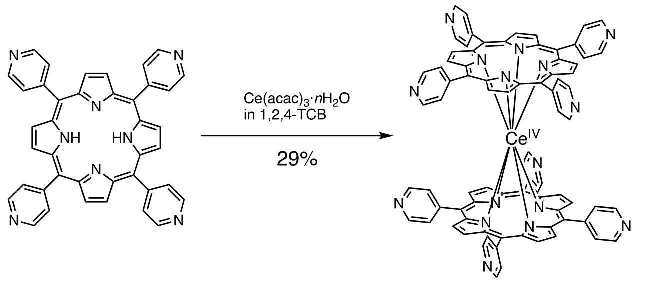

A mixture of 5,10,15,20-tetra(4-pyridyl)porphyrin (242 mg, 390 μmol) and $Ce(acac)_3 \cdot nH_2O$ (530 mg, 1.2 mmol (as $n = 0$)) in 1,2,4-TCB (32 mL) in a Schlenk flask was degassed by freeze-thaw method by 4 times. The resulting solution was refluxed under Ar stream for 13 hr at a bath temperature of 240 °C. The reaction mixture was cooled to room temperature and then poured into hexane (50 mL). The precipitate was filtrated and washed with hexane (30 mL × 2). The crude compound was purified by silica gel column chromatography (4ϕ × 13 cm, CH_2Cl_2: MeOH = 19 : 1 – 9 : 1, including 0.2% Et_3N) to afford a blackish brown solid. The crude material was washed with EtOH to afford the title compound as a brownish purple solid (79 mg, 29%). ^1H NMR (400 MHz, $CDCl_3$/TMS): δ = 9.45 (br, 17H), 8.61 (br, 8H), 8.34 (s, 21H), 6.35 (br, 8H). ESI-TOF-MS (positive) m/z = 1373.3 [**3** + H]$^+$, 1373.3 calcd for [**3** + H]$^+$.

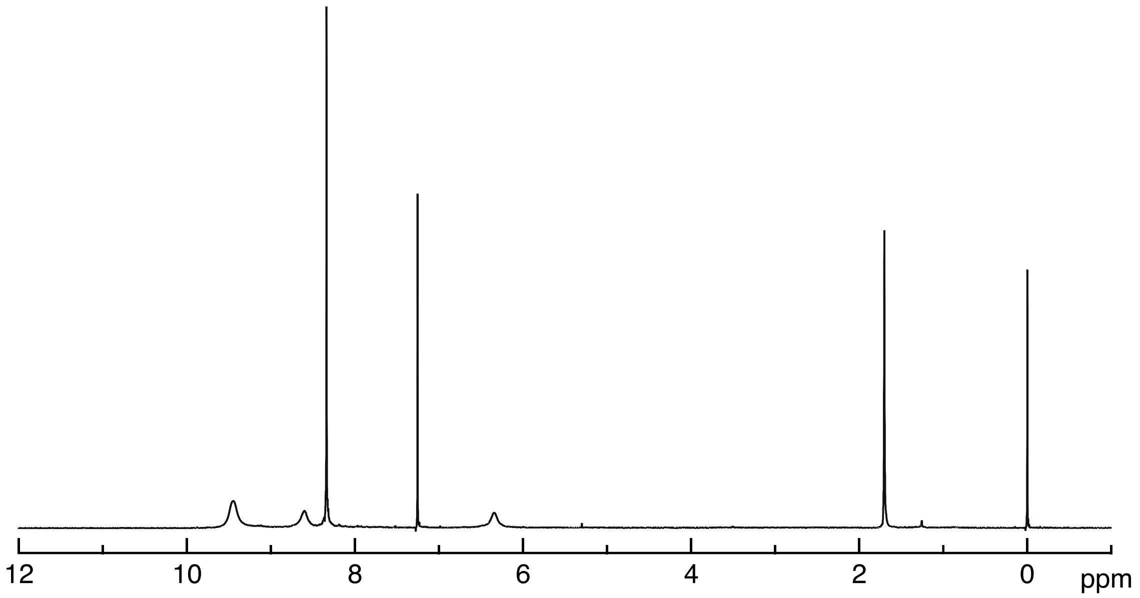

Figure S1. ^{1}H-NMR spectrum of bis(tetra(4-pyridyl)porphyrinato)cerium(IV) **3** in $CDCl_3$ at 20 °C.
